# Efficacy of PZT Sensors Network Different Configurations in Damage Detection of Fiber-Reinforced Concrete Prisms under Repeated Loading

**DOI:** 10.3390/s24175660

**Published:** 2024-08-30

**Authors:** Maria C. Naoum, Nikos A. Papadopoulos, George M. Sapidis, Maristella E. Voutetaki

**Affiliations:** 1 Laboratory of Reinforced Concrete and Seismic Design of Structures, Civil Engineering Department, School of Engineering, Democritus University of Thrace, 67100 Xanthi, Greece; mnaoum@civil.duth.gr (M.C.N.); nikpapad@civil.duth.gr (N.A.P.); gsapidis@civil.duth.gr (G.M.S.); 2 Architectural Engineering Department, School of Engineering, Democritus University of Thrace, 67100 Xanthi, Greece

**Keywords:** fiber-reinforced concrete (FRC), piezoelectric lead zirconate titanate (PZT), sensors network, electromechanical impedance (EMI), structural health monitoring (SHM), damage diagnosis

## Abstract

Real-time structural health monitoring (SHM) and accurate diagnosis of imminent damage are critical to ensure the structural safety of conventional reinforced concrete (RC) and fiber-reinforced concrete (FRC) structures. Implementations of a piezoelectric lead zirconate titanate (PZT) sensor network in the critical areas of structural members can identify the damage level. This study uses a recently developed PZT-enabled Electro-Mechanical Impedance (EMI)-based, real-time, wireless, and portable SHM and damage detection system in prismatic specimens subjected to flexural repeated loading plain concrete (PC) and FRC. Furthermore, this research examined the efficacy of the proposed SHM methodology for FRC cracking identification of the specimens at various loading levels with different sensor layouts. Additionally, damage quantification using values of statistical damage indices is included. For this reason, the well-known conventional static metric of the Root Mean Square Deviation (RMSD) and the Mean Absolute Percentage Deviation (MAPD) were used and compared. This paper addresses a reliable monitoring experimental methodology in FRC to diagnose damage and predict the forthcoming flexural failure at early damage stages, such as at the onset of cracking. Test results indicated that damage assessment is successfully achieved using RMSD and MAPD indices of a strategically placed network of PZT sensors. Furthermore, the Upper Control Limit (UCL) index was adopted as a threshold for further sifting the scalar damage indices. Additionally, the proposed PZT-enable SHM method for prompt damage level is first established, providing the relationship between the voltage frequency response of the 32 PZT sensors and the crack propagation of the FRC prisms due to the step-by-step increased imposed load. In conclusion, damage diagnosis through continuous monitoring of PZTs responses of FRC due to flexural loading is a quantitative, reliable, and promising application.

## 1. Introduction

Concrete is one of the most widely used materials globally, offering numerous benefits in structural applications, but two main challenges have yet to be fought. Firstly, plain concrete exhibits low tensile strength and poor fracture toughness, making it susceptible to cracking under tensile loads [1,2]. Secondly, the development of concrete cracks typically begins with microcracks that coalesce into macrocracks. The latter occurs due to the lack of coherence between the cement paste and aggregates in the interfacial transition zones when subjected to applied stresses. As these microcracks propagate, the concrete’s ability to withstand tensile loading diminishes significantly, ultimately leading to tensile failure [3,4].

Beyond the general application of conventional steel reinforcing bars, an alternative technique to control crack propagation and improve both tensional strength and post-cracking behavior of concrete is the addition of randomly dispersed discrete fibers in a well-designed and produced concrete matrix [5]. During the last decades, several kinds of fibers have emerged to face the demands of the construction industry [6,7,8]. Some of the most common fiber categories include metallic (steel and stainless steel), glass (silica and basalt), and synthetic fibers, such as polypropylene (PP), polyolefin (PO), polyester (PET), nylon, polyvinyl alcohol (PVA), polyethylene (PE), acrylic (PAN), aramid, and carbon [9,10]. However, until recently, most of the implementations using synthetic fibers were addressed to microfibers, substantially controlling and preventing the shrinking of cracks and elevating more ductile material behavior [11,12,13].

Additionally, an emerging trend is the application of macro-synthetic fibers in concrete mass to improve the post-cracking behavior of the material. Their long formation provides this merit as they can achieve to bridge the cracks in concrete, conveying stresses across the openings [14]. Improving concrete’s post-crack ductility, toughness, and macrocrack control at a low-width level prevents conventional steel reinforcement corrosion [15,16]. Furthermore, the composition of macro-synthetic ensures the avoidance of corrosion of the embedded fibers compared to metallic ones. Adding steel fibers can also reduce and delay cracking and increase the flexural, shear capacity, and ductility of RC beams subjected to monotonic or reverse cyclic loading [17,18,19,20,21]. Adding steel fibers to a concrete mix enhances the shear capacity by bridging the shear cracks, preventing a brittle failure mode [22,23].

In addition, the aging and corrosion of old, deteriorating infrastructures, combined with insufficient funding for their renovation, necessitate urgent structural integrity monitoring, making civil engineering applications a critical priority. Preventing structural deterioration or, even worse, sudden collapse could hinder both economic loss and severe casualties. Thus, the implementation of uninterrupted, real-time in situ surveillance to the detection of defects due to cracking or/and steel reinforcing yielding in critical elements in concrete structures, rendering a timely/early diagnosis, composes an optimized solution for structural health monitoring (SHM) [24,25,26].

The extant literature includes several studies investigating multiple methods of SHM. A non-destructive technique, X-ray microcomputed tomography, was employed to characterize the microstructural characteristics and mechanical properties of nano-reinforced cementitious composites [27]. Anjneya and Roy achieved SHM by examining changes in the dynamic properties of a six-story miniature structure. They proposed a comparative study over response surface methodology for damage detection using different modal parameters and the implementation of piezoelectric accelerometers. Vibration-based SHM has been used, and the structure’s acceleration response has been recorded, while other types of design experiments have been utilized to generate the response surface model [28].

Patil and Reddy presented a combination of vibration-based and non-destructive ultrasonic C-scan methods to identify, locate, and quantify the damage in impacted carbon composite plates. They also used piezoelectric fibers as sensors to provide information on the structural member’s global behavior and overall health condition. Different damage indices have been addressed to identify structural damage [29].

In the meantime, employing other SHM techniques that assess how fracture energy scatters within the microstructure has become essential for understanding how cracks spread and how damage distributes over the fracture surface [30]. These methods have demonstrated a significant correlation between the elastic and mechanical properties of fiber composites and can effectively describe the bonding interactions between fibers and the matrix [31,32].

In another relevant study, Gharehbaghi et al. propose a signal-based supervised methodology for detecting building deterioration and damage. The authors successfully applied the method to identify structures’ damage states according to various stress levels and the location and degree of damage. The methodology relied on baseline signals to extract the most sensitive features of any structure [33].

Khan et al. developed a data acquisition system for continuous monitoring that can be employed on reinforced concrete (RC) beams of existing structures using micro-electromechanical systems accelerometers as smart sensors. The damage to the beam was induced by applying the static load in increments of a fixed value. The time-series output data were received for each damage increment using excitations from an impact hammer [34].

Sokhangou et al. deployed modal curvature analysis to detect damages in UHPFRC beams [35], while Sapidis et al. presented a deep learning approach for autonomous compression damage identification in FRC [36]. Other researchers employed Fiber Bragg Grating sensors to detect the reinforcing bar’s slippage [37], the corrosion and loading force of steel bars [38,39], and the internal swelling pathologies in concrete blocks [40]. Furthermore, Perera et al. investigated the impact of sustained load and temperature on the performance of the Electromechanical Impedance Technique through Multilevel Machine Learning and FBG Sensors [41]. In addition, triaxial accelerometers were used for crack detection and localization in steel-fiber-reinforced self-compacting concrete [42]. 

Furthermore, smart piezoelectric materials, and especially piezoelectric lead zirconate titanate (PZT), and piezoresistive sensors, due to their advantageous properties due to piezoelectric effect operating as sensor and as actuator simultaneously, have been implemented in the electromechanical impedance (EMI) technique [43]. The first one excels in environments where detecting rapid changes or high-frequency signals is critical, while the piezoresistive sensors are often employed in devices that require precise and continuous measurement of static or slowly varying forces [44]. Several experimental and analytical studies demonstrated this arising and effective method in SHM applications in RC structures [45,46,47,48]. Recent research has indicated that the implantation of a network of PZT patches in regions of potential damage development substantially increases the efficacy and precision of these SHM methods to diagnose damage levels, providing reliable health monitoring in RC structural members [49,50,51,52]. Specifically, PZT-based SHM techniques have been extended by developing an advanced clustering approach that satisfactorily evaluates several measurements from the PZT network and diagnoses damage and debonding failure in RC members strengthened with fiber materials [53,54,55]. 

An advanced PZT-enabled EMI-based monitoring methodology has also been designed and verified by several monotonic and cyclic tests on RC small-sized specimens and full-scaled structural members. This recently developed real-time, wireless, portable SHM technique is called the “Wireless impedance/Admittance Monitoring System (WiAMS)”. It uses a network of small-sized PZT patches that are first excited by a predefined frequency response generated by the signal generator module of the custom-made, portable, and autonomous WiAMS device that consists of multiple modules. Next, this device’s peak detector module detects the sensors’ peak voltage response value, and their output voltage frequency response is received and recorded as a peak voltage value [56,57,58].

The literature review mentioned above reveals that despite several investigations concerning applying SHM techniques on concrete, limited studies deal with the monitoring procedures of FRC structural members [59,60]. The rapid progress in composite material science and construction innovation requires continuous monitoring and evaluation of structural conditions [52,61]. Thus, the applicability of a feasible real-time SHM technique in FRC with synthetic macro-fibers for detecting and predicting critical failures in FRC structures at the incipient damage stage is a topic of great importance.

This innovative research was motivated by the lack of extensive and reliable investigating methods to evaluate the tasks mentioned above. Furthermore, this research aims to determine the sensitivity of the developed PZT-enabled EMI-based method as far as the location, distance, and polarization direction of the employed PZT patches toward a potential failure.

The experimental part of the study includes three FRC specimens with synthetic fibers (SFRCs) and one plain concrete (PC) prismatic specimen subjected to a four-point bending under the impose of repeated loading. Thanks to the implementation of such a loading process, the PZTs’ voltage responses have been successfully acquired at different levels of the imposed loading and corresponding structural health conditions (loading states). In this way, the specimens’ structural integrity could be assessed. In addition, the damage diagnosis was examined using a WiAMS device and PZT patches were installed in different locations.

The higher ductility of SFRC presents challenges compared to the PC in investigating the efficacy of different PZT network configurations. It allows additional loading steps on unreinforced elements and limits crack propagation (width, length, and depth). This reduction in crack propagation helps minimize and control the uncertainties in PZT measurements before the onset of a catastrophic brittle failure. The damage indices, Root Mean Square Deviation (RMSD), and Mean Absolute Percentage Deviation (MAPD) were used in this scope.

Furthermore, the threshold Upper Control Limit (UCL) was also employed to examine the sensitivity and evaluate each PZT patch’s efficacy, sifting the extracted RMSD and MAPD values.

## 2. Experimental Program

### 2.1. Materials and Preparation

The details of the mix proportions and the mechanical properties of the concrete used in this study are provided in Table 1. The coarse aggregate included crushed stone aggregates with a maximum size of 16 mm, while the fine aggregate consisted of high fineness modulus crushed sand. The cement used conformed to the requirements of standard EN206 [62].

The macro synthetic fibers used in SFRC mixtures are made of polyolefin. They have a continuous corrugated appearance, which enhances bond characteristics with concrete due to surface anchorage. Table 2 presents the details and properties of the fibers. The fibers were added directly to the fresh concrete batch at a proportion of 5 kg per 1 m^3^ of concrete.

**Figure 1:**
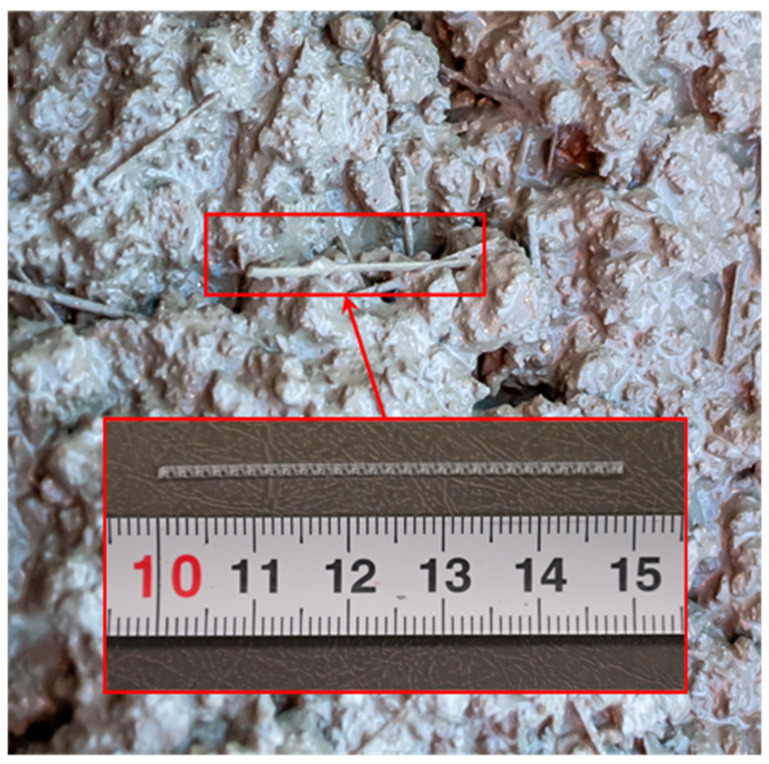
Fresh SFRC, including fibers.

Careful consideration was given when incorporating the fibers into the pan to ensure even distribution and maintain the flowability of the fresh SFRC mixture. The macro synthetic fibers were gradually added by hand in small quantities during the mixing process to prevent clumping. The mixture was stirred progressively to achieve a uniform consistency, enhance workability, and ensure a homogeneous distribution of the fibers, thereby preventing their segregation in the fresh SFRC, Figure 1. Finally, the prepared SFRC was poured into the molds and properly vibrated.

### 2.2. Tests and Specimens

**Figure 2:**
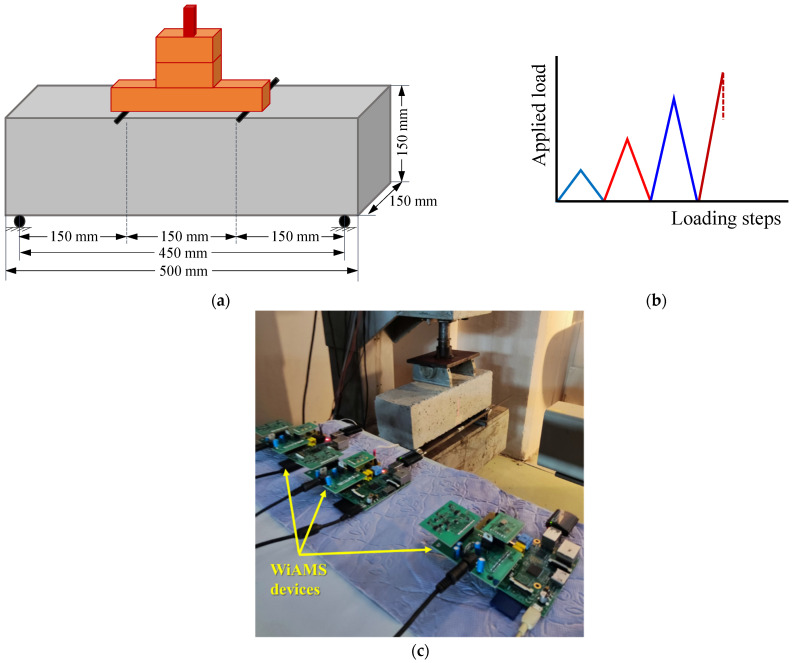
(**a**) Test set up, (**b**) loading sequence, and (**c**) WiAMS devices.

The test program consisted of a PC and three SFRC prismatic specimens with dimensions 500 mm (length) × 150 mm (height) × 150 mm (width). Specimens were tested under a standard four-point bending configuration, as shown in Figure 2a. The concrete specimens were supported on hardened steel rollers at the two supports over an L = 450 mm span. Two-line loads were applied at a 75 mm distance on both sides from the middle of the specimen’s top surface with two hardened steel rollers. The tests were performed employing a servo-controlled hydraulic machine, and their performance was examined in a four-point bending test as per ASTM C78 [63] standard for obtaining the fracture response of concrete. 

The tested specimens were subjected to repeated load, including loading, unloading, and reloading, under varying load levels per loading step based on the estimated maximum flexural strength. The loading sequence is shown schematically in Figure 2b, and Figure 2c presents the WiAMS devices installed on the PZTs. In the final step, the specimens were loaded until failure, as the developed flexural stress reached the ultimate load-carrying bending capacity, causing a pure flexural fracture at the mid-span. The max load rate at each cycle and the percentage of the flexural max strength are detailed in Table 3. The load rate at each cycle was approximately 0.9–1.2 MPa/min.

The flexural tests were performed in force control, considering the estimated maximum load for each examined loading step. During the tests, load and deflection measurements were continuously monitored and recorded. Further, full-field surface displacements from the specimens were obtained using Laser Extensometers (LE), LE-05 model from Εpsilontech International. 

**Figure 3:**
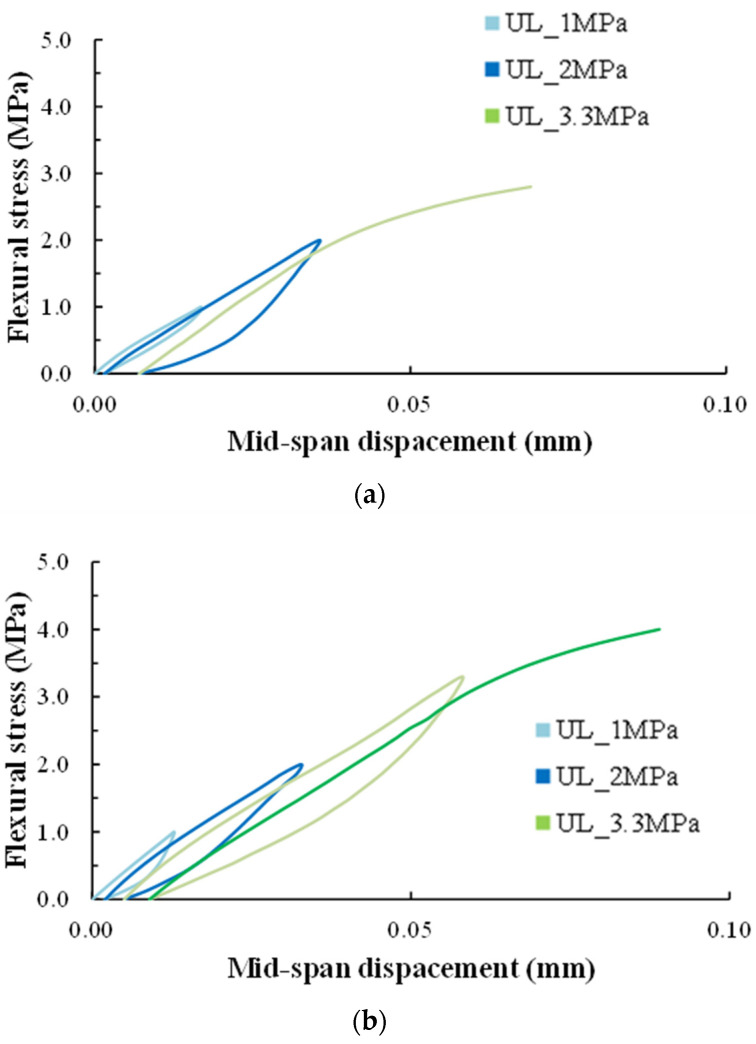
Experimental behavior of the (**a**) PC and (**b**) SFRC specimens.

Diagrams of Figure 3 demonstrates the typical experimental behavior of a PC and SFRC specimen in terms of flexural stress versus mid-span deflection, respectively. The values of the flexural stress were calculated according to Equation (1):

(1) $$\sigma_{f}=\frac{F\cdot L}{b\cdot h^{2}}$$

where *σ_f_* = stress in outer fibers at the midpoint (MPa), *F* = Applied load (N), *L* = Support span (=450 mm), *b* = Width of test beam (=150 mm), and *h* = height of tested beam (=150 mm).

**Figure 4:**
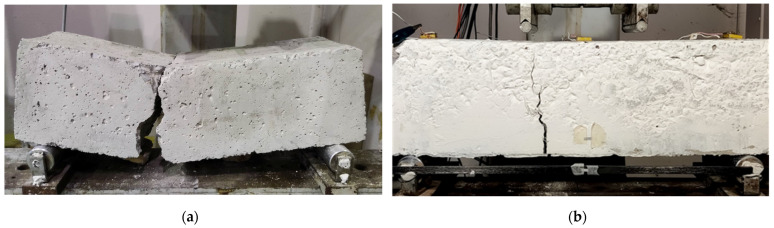

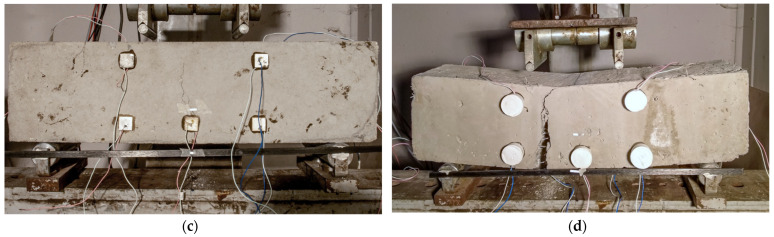
Cracking pattern of tested specimens at failure: (**a**) Specimen 1; (**b**) Specimen 2; (**c**) Specimen 3; (**d**) Specimen 4.

The cracking patterns of the specimens at failure are displayed in the photographs of Figure 4a–d.

### 2.3. SHM Method and PZT Sensor’s Network

The present experimental study used a wireless SHM and damage diagnosis system based on the EMI method. The EMI measurements were implemented through custom-made devices (WiAMS) using PZT patches. The effectiveness of WiAMS devices in evaluating the structural integrity of PC and SFRC specimens under the subjection of a repeated loading four-point bending test was investigated.

The well-known merits of the PZT patches, especially their capability to act as sensors subjected to vibration by an amplified harmonic excitation voltage signal and simultaneously as sensors receiving the reflected waves in terms of electrical impedance frequency response, were also utilized. Furthermore, multiple configurations of PZT patches regarding their bonding condition, location, and polarization direction were investigated to evaluate each combination’s efficiency. 

The dimensions of the used PZT sensors were 20 × 20 × 5 mm. After casting and demolding the hardened FRC specimens, multiple configurations of PZT sensors were meticulously epoxy- and cement-paste-bonded on the surface of the prisms at different positions relative to the expected location of the potential imminent failure. Thus, the sensors’ site related to their distance from the critical crack and the influence on the SHM technique’s efficiency is also examined. 

It is essential to consider several factors that can influence the sensor’s performance to adjust the configurations of a PZT sensor network for detecting defects with varying characteristics, such as crack depth, width, and length. A short discussion of the implemented configurations and their respective impacts on defect detection is presented below:

- Surface Epoxy-Bonded PZT Patches

The most common setup involves PZT patches that are surface-bonded directly to the structure using epoxy resin. This configuration ensures good mechanical coupling between the sensor and the specimen, a critical factor in increasing the efficacy of detecting structural anomalies.

- Epoxy-Inclined PZT Patches in Grooved Notches

Another configuration involves positioning the PZT patches inclined within specially grooved notches on the surface. This approach may enhance the interaction between the PZT sensors and the host structure by reducing damping.

- Cement Paste-Coated PZTs

In this setup, PZT sensors are coated with cement paste before being bonded to the surface of the specimen. The cement coating serves a dual purpose; it protects the sensors in real applications and also influences the bonding and signal transmission process parameters between the sensor and the host structure.

- The angle of PZT Polarization Relative to Crack Direction

This study also investigates the effect of aligning the PZT polarization at various angles to the direction of the anticipated crack. In particular, some PZT sensors are molded at a predefined angle of around 45 degrees and then coated with cement paste.

Adjusting PZT sensor configurations involves carefully considering the crack characteristics of the intended monitoring. Factors such as sensor placement, orientation, coating, and bonding methods can all be tailored to enhance the detection of specific crack properties like depth, width, and length. By optimizing these configurations, the efficacy of the PZT network in identifying and characterizing structural defects could be achieved. The depth and the width of cracking could be achieved more efficiently by the vertically positioned PZTs. At the same time, determining the crack length demands a network of PZTs aligned with its propagation. 

The aforementioned implemented configurations were investigated to determine their efficiency regarding the cracking characteristics. Thus, for the crack depth, the sensitivity of surficial epoxy-bonded PZTs to crack depth can be enhanced by optimizing the placement of sensors closer to areas expected to experience higher stress concentrations. Inclined PZTs can be strategically positioned to focus on detecting defects that propagate deeper within the material. The angle of inclination can be adjusted depending on the expected depth of the cracks to maximize sensitivity.

**Figure 5:**
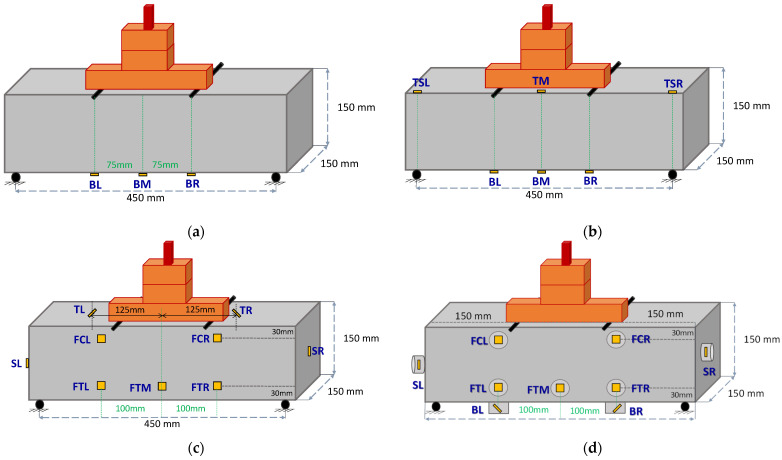
Configuration and positions of the used PZT patches mounted to the FRC prisms: (**a**) Specimen 1; (**b**) Specimen 2; (**c**) Specimen 3; (**d**) Specimen 4.

Characteristics and positions of all the installed PZT sensors for all tested specimens are presented in Figure 5. The notation of each PZT patch according to its position is as follows (see also Figure 4):

*Configuration of specimen 1 (PC)*

- Three PZT patches were epoxy-bonded to the bottom surface of the prism; one on the middle of the surface (BM: Bottom Mid), one on the right side (BR: Bottom Right), and one on the left side (BL: Bottom Left), both at a distance of 75 mm from the middle of the specimen, directly opposite to the two loading points.

*Configuration of specimen 2 (SFRC)*

- Three PZT patches were epoxy-bonded to the bottom surface of the prism; one on the middle of the surface (BM: Bottom Mid), one on the right side (BR: Bottom Right), and one on the left side (BL: Bottom Left), both at a distance of 75 mm from the middle of the specimen, directly opposite to the loading points.
- One PZT patch was epoxy-bonded in the middle of the top surface of the prism (TM: Top Mid).
- Two PZT patches were epoxy-bonded to the left and right on the top surface, directly opposite each support (TSL: Top Support Left) and (TSR: Top Support Right).

*Configuration of specimen 3 (SFRC)*

- One PZT patch was epoxy-bonded in the middle of the facade of the specimen and the tension zone (FTM: Facade Tension Middle).
- Two PZT patches were epoxy-bonded on the right side of the façade of the prism at a 100 mm distance from the middle of the specimen; one in the tension zone (FTR: Facade Tension Right) and one in the compression zone (FCR: Facade Compression Right).
- Two PZT patches were epoxy-bonded on the left side of the prism facade, positioned 100 mm away from the specimen’s center; one patch was placed in the tension zone (FTL: Facade Tension Left) and the other in the compression zone (FCL: Facade Compression Left).
- Additionally, two PZT patches were epoxy-bonded at the mid-height and mid-width of each end-free side of the prism; one on the right side (SR: Side Right) and one on the left side (SL: Side Left). Two PZT patches were inclined epoxy-bonded at a distance of 125 mm left and right from the mid-point of the top surface (TIL: Top Inclined Left) and (TIR: Top Inclined Right), respectively.

*Configuration of specimen 4 (SFRC)*

- A single cement paste-coated PZT patch was bonded to the center of the specimen’s facade in the tension zone (FTM: Facade Tension Middle).
- Two cement paste-coated PZT patches were attached to the right side of the prism’s facade, 100 mm from the specimen’s center; one was placed in the tension zone (FTR: Facade Tension Right) and the other in the compression zone (FCR: Facade Compression Right).
- Two cement paste-coated PZT patches were similarly bonded to the left side of the prism’s facade, also 100 mm from the specimen’s center; one in the tension zone (FTL: Facade Tension Left) and one in the compression zone (FCL: Facade Compression Left).
- On the bottom of the prism, two cement paste-coated PZT patches were bonded, one on the right side (BR: Bottom Right) and one on the left side (BL: Bottom Left), each positioned 100 mm from the specimen’s center. Both sensors (BL and BR) were cast at a predefined 45° angle.
- Finally, two cement paste-coated PZT patches were attached at the mid-height and mid-width of each side of the prism; one on the right side (SR: Side Right) and one on the left side (SL: Side Left).

### 2.4. Quantitative Assessment of Damage

It is acknowledged that a visual comparison of the differences between the healthy response and every subsequent one in the next damage states could not be competent enough to determine the structural integrity. Therefore, the application of a sufficient quantitative assessment of damage is essential.

For this purpose, the traditionally used damage indices of RMSD and MAPD are applied. The expressions of both indices are presented below in Equations (2) and (3):

(2) $$RMSD=\sqrt{\frac{\sum_{1}^{Μ}{\left(\left|{V_{p}(f_{r})}_{D}\right|-\left|{V_{p}(f_{r})}_{0}\right|\right)}^{2}}{\sum_{1}^{Μ}{\left(\left|{V_{p}(f_{r})}_{0}\right|\right)}^{2}}},$$

(3) $$MAPD=\frac{1}{M}\sum_{r=1}^{M}\left|\frac{{V_{p}\left(f_{r}\right)}_{D}-{V_{p}\left(f_{r}\right)}_{0}}{{V_{p}\left(f_{r}\right)}_{0}}\right|,$$

where ${V_{p}(f_{r})}_{0}$ is the voltage output signal extracted from the PZT sensor at the specimen’s healthy pristine state of the specimen in a specific frequency $f_{r}$, ${V_{p}(f_{r})}_{D}$ is the corresponding voltage output signal measured from the same PZT at any damage level, and M is the number of measurements in the frequency band of 10–250 kHz.

Furthermore, a data quality threshold index was also introduced to evaluate the sensitivity of each PZT sensor. Generally, thresholds may be established in several different ways, either manually, based on reasonable levels, or automated based on a calculation such as the mean or median of data. This study employed the Upper Control Limit (UCL) as a numeric representation of the acceptable measurement limit. Thus, every index value exceeding the computed threshold indicates a potential abnormality in the effective monitoring area of the particular patch. First, the UCL threshold was applied to a set of 20 consecutive measurements in the pristine condition of each specimen for every single PZT patch. Then, UCL was calculated as below in Equation (4):

UCL = μ + 3σ,(4)

where μ represents the mean value of the impedance measurements at the pristine condition and σ is the standard deviation at the same condition.

## 3. Results and Discussion

### 3.1. Analysis of Voltage and Indices

The experimented specimens showed the characteristic flexural response and brittle failure, as they were designed and expected according to the test method. Additionally, the SFRC specimens demonstrated higher bearing capacity compared to the PC, as shown in Figure 3. Typically, the critical flexural crack occurred at the mid-span and perpendicular to the longitudinal axis of the specimen (see also the cracking pattern of each specimen at failure in the photographs of Figure 4). 

The PZT sensors were separated into groups according to their polarization orientation toward the critical crack at failure or/and an axis of symmetry to assess the efficacy of each PZT’s configuration. Thus, the groups of the PZTs are comprised as below:

*Specimen 1*

(a) BL and BR and (b) BM and BL sensors.

*Specimen 2*

(a) BL and BR, (b) BM and TM, and (c) TSR and TSL sensors.

*Specimen 3*

(a) FTL, FTR, and FTM, (b) FCL and FCR, (c) TIL and TIR, and (d) SL and SR

*Specimen 4*

(a) FTL, FTR, and FTM, (b) FCL and FCR, (c) BL and BR, and (d) SL and SR

Analyzing the values of the selected indices is an attempt to identify similarities and differences in the responses of the grouped PZT sensors. The following sections present and discuss typical responses of the PZT voltage frequency responses and the RMSD and MAPD index ratios for each PZT sensor for each specimen.

#### 3.1.1. Specimen 1

**Figure 6:**
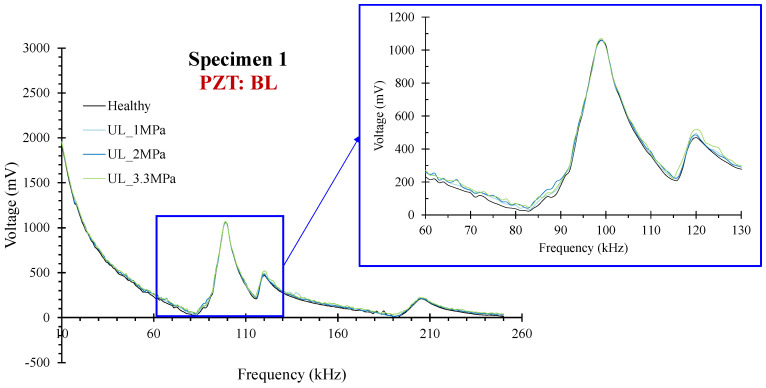
Specimen 1: Typical voltage frequency response of the PZT sensor BL.

**Figure 7:**
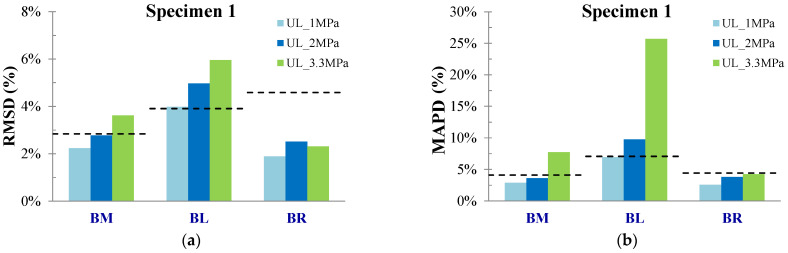

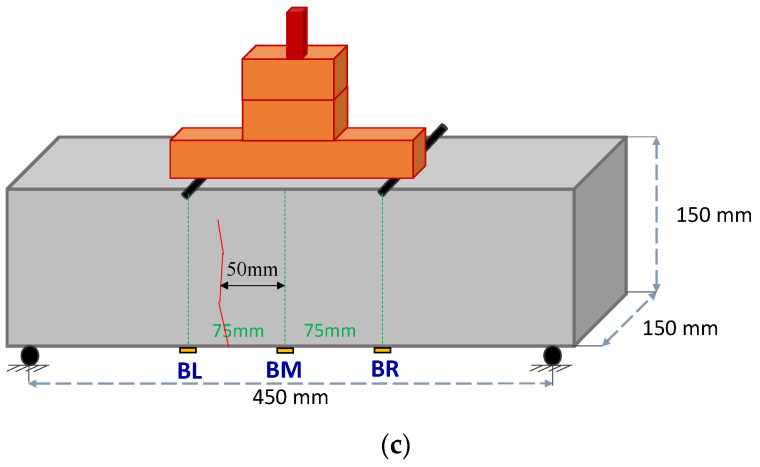
Damage assessment measurements considering (**a**) RMSD and (**b**) MAPD indices values of all PZTs of Specimen 1, and (**c**) cracking pattern of Specimen 1.

PZTs BL, BR, and BM were positioned at the bottom surface of the specimen at the left, right, and middle of the mid-span, respectively. Figure 6 depicts the voltage responses of the healthy response and the subsequent ones in the following loading states of PZT BL. In specimen 1, the critical failure was formed in the mid-span at a distance of around 50 mm left from the mid-point Figure 7c. Therefore, PZT BL showed higher ratios of damage indices among the three bottom-placed sensors Figure 7. The noticeable point in this specimen is that the threshold UCL, which is depicted in the diagrams with a black dashed line, trimmed the ratios of PZTs BR, and BM, in alignment with the actual development of the cracking pattern. Thus, although there are appreciable indices ratios at all the loading states for BR and BM, the interpretation of the data is that these values are statistically insignificant, and further, no significant abnormalities are expected to be met in their monitoring areas at these states. Contrariwise, the RMSD ratios of PZT BL exceed the UCL value slightly at loading level UL_2 MPa, and further at the failure state, demonstrating the diagnosis of the damage. Moreover, the ratio of RMSD index PZT BM at failure also exceeds the UCL threshold but in a lower percentage than PZT BL, a fact that verifies the closer proximity of the failure to PZT BL. 

Furthermore, the trend of the MAPD values Figure 7b followed the abovementioned comments but in higher ratios, a condition that demonstrates the higher sensitivity of the scalar index compared to RMSD.

#### 3.1.2. Specimen 2

**Figure 8:**
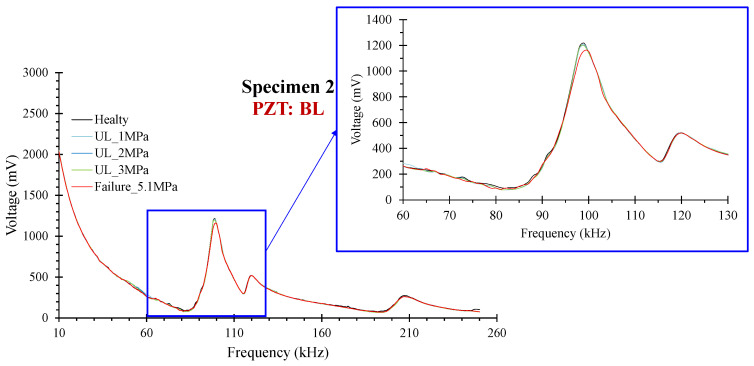
Specimen 2: Typical voltage frequency response of the PZT sensor BL.

**Figure 9:**
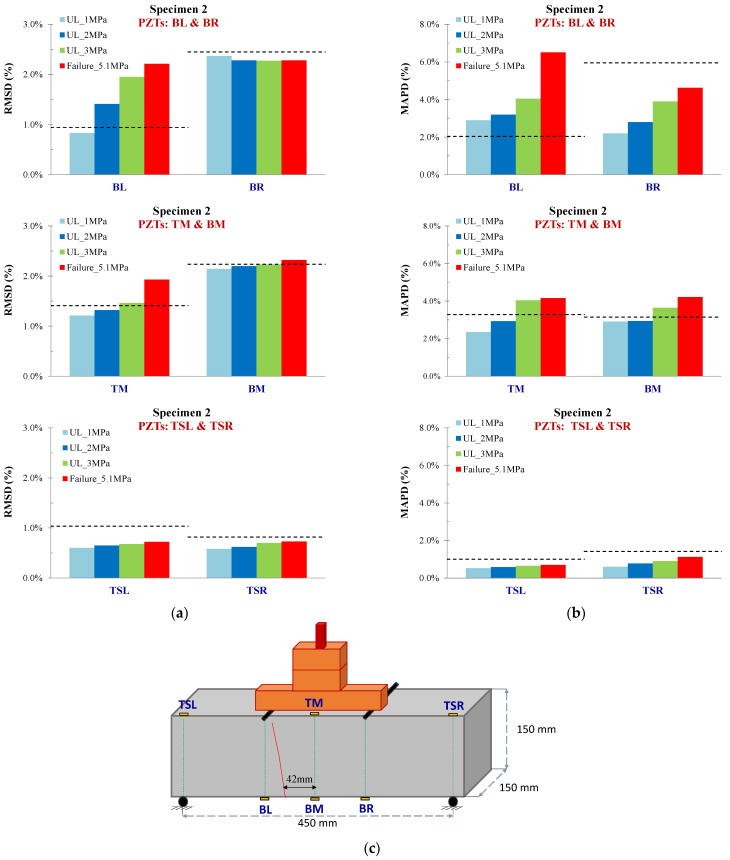
Damage assessment measurements considering (**a**) RMSD and (**b**) MAPD indices values of all PZTs of Specimen 2, and (**c**) cracking pattern of Specimen 2.

PZTs BL, BR, and BM were positioned at the bottom surface of the specimen at the left, right, and middle of the mid-span, respectively. Figure 8 shows the voltage responses for all the loading states of PZT BL. In specimen 2, the critical failure was developed in the mid-span at a distance of approximately 42 mm left from the mid-point. Therefore, PZT BL showed higher ratios of damage indices among the three bottom-placed sensors and the TM (Top Mid). Similarly to specimen 1, the noticeable point in this specimen is that the threshold UCL (depicted in a black dashed line in the diagrams of indices) sifted the ratios of PZTs BR, BM, TSL, and TSR following the actual development of the cracking pattern, as depicted in Figure 9. Thus, although there are considerable RMSD ratios at some loading states, especially for BR and BM, the data analysis elucidates that these values are statistically insignificant, and further, no significant abnormalities are expected to be met in their monitoring areas at these states. Contrariwise, the RMSD ratios of PZT BL exceed the UCL value slightly at the primary loading levels UL_1 MPa, UL_2 MPa, and further at UL_3 MPa and the failure state, demonstrating the prompt diagnosis of the damage. Moreover, the ratio of RMSD index PZT BM at failure also exceeds the UCL threshold but in a lower percentage than PZT BL, a case that verifies the closer proximity of the failure to PZT BL. Furthermore, the trend of the MAPD values also prescribed the abovementioned comments, but in higher ratios, a fact demonstrating the scalar index’s higher sensitivity than the RMSD.

#### 3.1.3. Specimen 3

**Figure 10:**
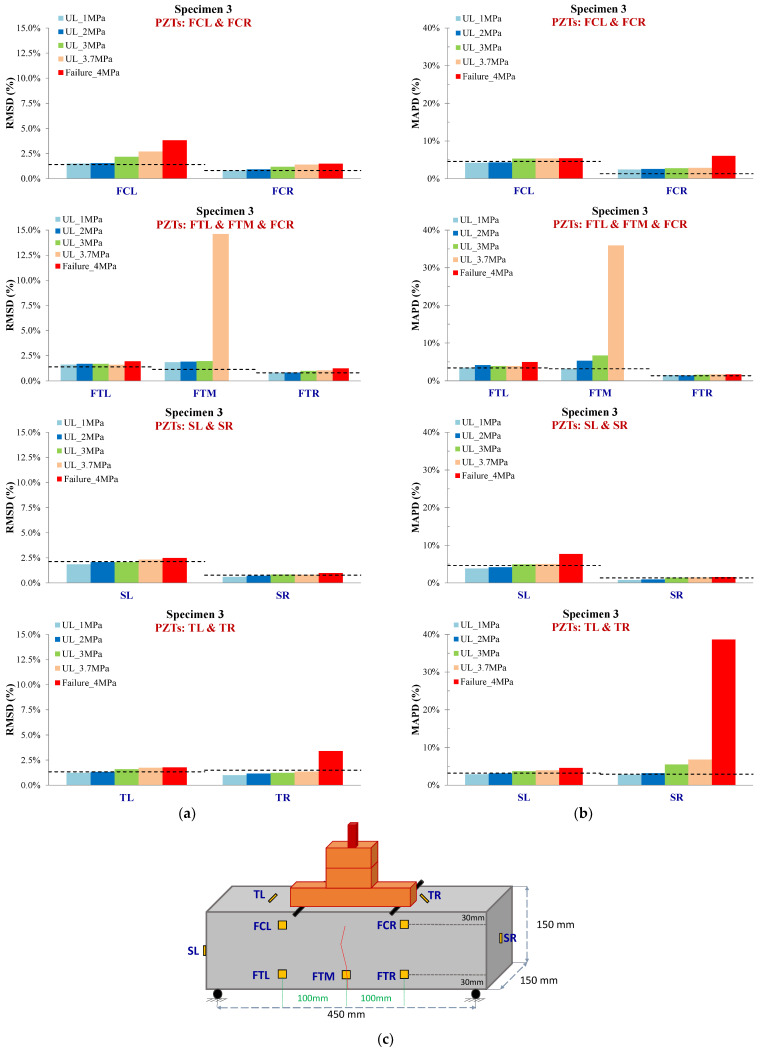
Damage assessment measurements considering (**a**) RMSD and (**b**) MAPD indices values of all PZTs of Specimen 3, and (**c**) cracking pattern of Specimen 3.

In Specimen 3, the critical failure occurred exactly at the midpoint of the mid-span. PZTs TL and TR were designed to be positioned at a predefined angle of 45° with an intersecting polarization orientation relative to the anticipated and ultimately occurred critical failure. Finally, at the end of the experiment, the calculated constructed angles were 40.5° and 36.5°, respectively, due to the construction’s imperfections. Hence, this agent influenced the total designed monitoring efficacy of each PZT patch due to the lengthening of the distance between the patch and the finally formed crack, reducing the monitoring range in the crack’s area. Moreover, the previous comment is verified by the presented results of scalar indices in Figure 10. Thus, PZT TL exhibited UCL-exceeded values at earlier loading states than PZT TR.

**Figure 11:**
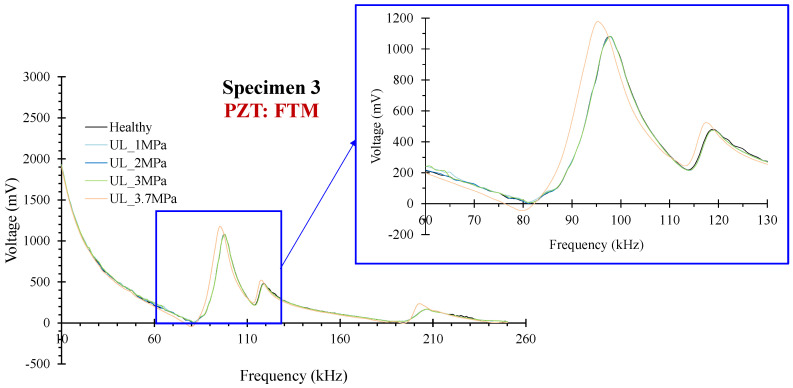
Specimen 3: Typical voltage frequency response of the PZT sensor FTM.

Furthermore, PZTs FCL and FCR revealed UCL-exceeded values at earlier loading states and generally a similar trend. In addition, PZTs FTL and FTR showed slightly UCL-transcended values at almost all the loading states. On the contrary, PZT FTM at pre-failure loading state UL_3.7 MPa exhibited an abrupt increment of the RMSD ratio, a pre-runner indication of the forthcoming failure, which finally intersected the substrate area of the patch, leading to its malfunction at the last set of measurements. A remarkable point, in this case, is that the last pre-failure measurement was acquired at 92.5% of the maximum loading. Additionally, PZTs SL and SR showed some minor UCL-exceeded values, especially after the occurred failure. Figure 11 shows the voltage discrepancy of the healthy response and every subsequent one in the following loading states for PZT FTM.

All the PZTs more or less exhibited the same trend with higher ratios for scalar index MAPD withal. The only significant exception is the ratio of PZT TR at the failure state, which exhibited an extremely high percentage compared to the relevant RMSD one.

#### 3.1.4. Specimen 4

**Figure 12:**
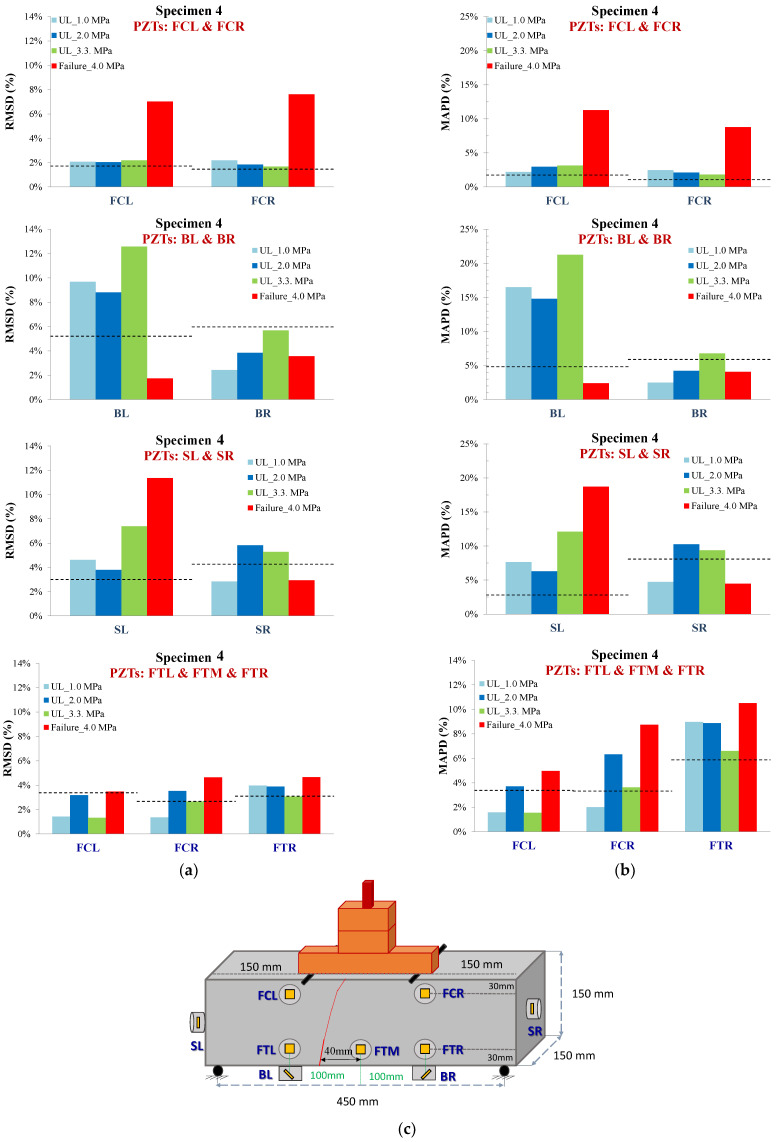
Damage assessment measurements considering (**a**) RMSD and (**b**) MAPD indices values of all PZTs of Specimen 4, and (**c**) cracking pattern of Specimen 4.

For specimen 4, the critical failure was formed at a distance of 40 mm left of the mid-point of the mid-span. Further, the essential critical results extracted from the specimen are the performance of PZTs BL and BR, which were coated at an inclined predefined angle of 45^o^. The PZTs were mounted with an intersected polarization direction to the expected and finally formed critical failure. Figure 12 shows the voltage responses in terms of the frequency range for all the loading states for PZT BL.

**Figure 13:**
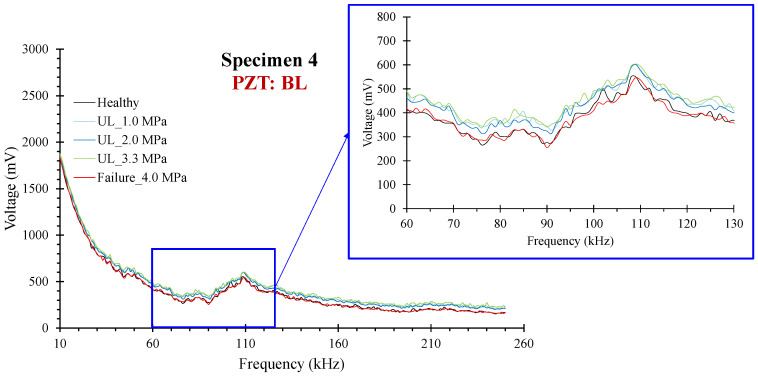
Specimen 4: Typical voltage frequency response of the PZT sensor BL.

In specimen 4, PZT BL showed UCL-exceeded values from the early loading states till the final failure. Furthermore, PZTs FCR and FCL showed a significant change at the final loading state with an abrupt alteration of the RMSD ratio. Moreover, PZT FCR exhibited a slightly higher final ratio. The latter is probably caused due to the lateral turn of the crack’s propagation to the right direction in the upper section of the specimen, as shown in Figure 13c. 

Further, PZTs FTR, FTM, and FTL showed slightly increased UCL-transcended RMSD values at early loading states and higher values at failure. Moreover, PZT FTL, and FTM showed more elevated than the relevant PZT FTR, a condition verified by the crack’s final location, which occurred between FTL and FTM.

Additionally, PZTs SR and SL were bonded to the outer free-end sides of the specimen, positioned in a vertical polarization direction to the potential failure. The primary purpose of locating the PZTs at the free-ends was to ensure monitoring of the extreme areas in case of an unexpected and out-of-the-mid-span critical failure. Despite that, PZT SL seemed to have been influenced by the position of the failure. PZT SL exhibited UCL-exceeded RMSD values in the third and final loading state. 

Conversely, PZT SR also showed increased RMSD values considering that the failure was formed at a distance of limited monitoring capability; the application of the UCL threshold sifted the significance of its measurements. 

All the PZTs’ values of the scalar index MAPD followed a similar trend with RMSD withal as shown in Figure 13b.

Synoptically, PZTs closer to critical failure in comparison to the relevant symmetric of their configuration seem to give an alarm at an earlier damage state and have greater values of RMSD and MAPD indices. All the left PZTs seemed more sensitive and efficient than their symmetric right ones. It is of high importance that the adjacent sensors responded satisfactorily to the failure, as there were no obvious damages at this state.

## 4. Conclusions

This experimental study investigated the sensitivity in damage diagnosis of an EMI-based system using multiple PZT sensor configurations in SFRC prisms. The principal pillars of this study were the selection of a four-point testing set-up and the use of SFRC specimens. The combination of both parameters developed a case study investigating the formation of one significant cracking in a particular specimen area (mid-span). The abovementioned condition assists in examining the sensitivity of the applied monitoring device and further evaluating the efficiency of the PZT sensors regarding their location and implemented configuration. Furthermore, the formation of one major crack and the acquired impedance measurements in a release condition limited the impact of the induced stress state due to the imposed load or concentrated stresses developed due to damage. In addition, damage diagnosis of SFRC specimens subjected to four-point bending testing under repeated loading was attempted using a custom-made EMI-based SHM system and a network of PZT sensors. The main contributions and conclusions of this work are recapitulated as follows:

- SFRC specimens exhibited higher values of loading compared to the PC one. Further, SFRC specimens led to a more controlled brittle failure with a repairable cracking pattern, compared to the PC, where the specimen was separated into two individual sections.
- The SHM system’s effectiveness in damage diagnosis in a four-point bending test of SFRC specimens using voltage responses of specially mounted piezoelectric sensors has been experimentally investigated. Four different regions of PZTs application have been examined: (a) on the front face, (b) at the bottom surface, (c) at the top surface, and (d) at the free-end sides of the specimens.
- Damage diagnosis has been attempted using values of the known statistical RMSD and MAPD indices to improve the efficiency and accuracy of the applied technique.
- UCL threshold value has been implemented to enhance the accuracy of the measurements, evaluating the significance of each PZT patch individually. Hence, determining each patch’s sensitivity is crucial to establishing a reliable SHM monitoring technique by sifting the extracted indices.
- Voltage responses of the PZT acquired from the test measurements showed obvious discrepancies between the healthy state and the examined loading levels for each specimen. These differences indicate the presence of a potential abnormality. The implementation of the UCL threshold assists in determining that values below UCL are judged as insignificant, values slightly higher than the UCL need further examination, and considerably higher values constitute indications of potential damage.
- It has been found that approaching the final failure, the indices’ values of the contiguous PZTs are rationally trending upwards. Thereupon, most of the examined PZTs’ responses exhibited such a performance.
- It is emphasized that the indices’ values of the PZT BL mounted to the bottom surface of the specimens at a predefined angle of around 45° show elevated monitoring performance. Moreover, there are promising indications that the monitoring efficiency of the PZT sensor is inseparably linked with its sufficient positioning to the examined host structure. In addition, the closer to 90° is the angle formed between the formed crack and the polarization direction of the PZT, the more the monitoring efficiency increases. Therefore, the acquired measurements of the PZT sensors mounted to such locations could help enhance damage diagnosis performance and constitute indicators of prediction of the forthcoming failure at early damage stages.
- In the case of specimen 4, and following the previous conclusion, PZT SL mounted to the left side of the prism showed better results due to the angle of the crack formed, which is slightly inclined towards the left span of the prism.
- This study’s results concentrated on evaluating the efficiency of the PZT sensor’s network configuration. Further relevant investigation is needed to examine similar and multiple configuration types to enhance the proposed method’s reliability.
- Additional experiments are necessary to enhance the standardization procedures of the applied SHM technique. Furthermore, it is important to conduct further research using the proposed SHM technique on various structural elements and materials to build a comprehensive database. By applying statistical and data analysis to this database, a procedure similar to vulnerability curves can be developed, allowing for the baseline assessment of the current integrity of structural elements. Thus, the proposed method could be applied to existing structures with essential impact. Furthermore, combining NDT methods, SHM technique, and Finite Element simulations can also be tools for determining the current structural integrity.

## Figures and Tables

**Table 1:** Concrete properties.

Mix Proportion (Cement: Water: Fine Aggregate: Coarse Aggregate)	Density (Kg m^−3^)	Compressive Strength (MPa)	Young’s modulus (GPa)	Modulus of Rupture (MPa)			
				Spec. 1	Spec. 2	Spec. 3	Spec. 4
1:0.52:2.4:2	2355	46	32	3.3	5.1	4.0	4.0

**Table 2:** Properties of the macro synthetic fibers.

Type (Name)	Length (mm)	Equivalent Diameter (mm)	Young’s Modulus (GPa)	Tensile Strength (MPa)
SikaFiber Force 50	50	0.715	6	430

**Table 3:** Max load rate at each cycle and the percentage of the flexural max strength.

	Cycle	Max. Load/Cycle (MPa)	Ultimate Damage Level (UL)	Percentage of the Flexural Max Strength
Specimen 1	1	1.0 MPa	UL_1 MPa	30%
	2	2.0 MPa	UL_2 MPa	60%
	3	3.3 MPa	Failure_3.3 MPa	Failure (max strength)
Specimen 2	1	1.0 MPa	UL_1 MPa	20%
	2	2.0 MPa	UL_2 MPa	39%
	3	3.0 MPa	UL_3.0 MPa	59%
	4	5.1 MPa	Failure_5.1 MPa	Failure (max strength)
Specimen 3	1	1.0 MPa	UL_1 MPa	25%
	2	2.0 MPa	UL_2 MPa	50%
	3	3.7 MPa	UL_3.7 MPa	93%
	4	4.0 MPa	Failure_4.0 MPa	Failure (max strength)
Specimen 4	1	1.0 MPa	UL_1 MPa	25%
	2	2.0 MPa	UL_2 MPa	50%
	3	3.3 MPa	UL_3.3 MPa	83%
	4	4.0 MPa	Failure_4.0 MPa	Failure (max strength)

## Data Availability

The data presented in this study are available upon request from the corresponding author.
